# The Modulation of Septic Shock: A Proteomic Approach

**DOI:** 10.3390/ijms251910641

**Published:** 2024-10-03

**Authors:** Patrícia Terra Alves, Aline Gomes de Souza, Victor Alexandre F. Bastos, Eduarda L. Miguel, Augusto César S. Ramos, L. C. Cameron, Luiz Ricardo Goulart, Thúlio M. Cunha

**Affiliations:** 1 Laboratory of Nanobiotechnology, Institute of Biotechnology, Federal University of Uberlândia, Uberlândia 38402-022, MG, Brazilthcunha@yahoo.com.br (T.M.C.); 2 Department of Medical Imaging, Hematology and Oncology, Ribeirão Preto Medical School, University of São Paulo, Ribeirao Preto 14040-900, SP, Brazil; alinegs@usp.br; 3 Laboratory of Biochemistry, Institute of Biotechnology, Federal University of Uberlândia, Uberlândia 38408-100, MG, Brazil; victor.bastos@ufu.br; 4 School of Medicine, Federal University of Uberlândia, Uberlândia 38408-100, MG, Brazil; draeduardalm@gmail.com (E.L.M.); augustocips@gmail.com (A.C.S.R.); 5 Arthritis Program, Schroeder Arthritis Institute, Krembil Research Institute, University Health Network, Toronto, ON M5T 0S8, Canada; lc.cameron@uhn.ca; 6 Lorraine Protein Biochemistry Group, Graduate Program in Neurology, Gaffrée e Guinle University Hospital, Rio de Janeiro 20270-004, RJ, Brazil

**Keywords:** monocytes, secreted proteins, hypotension, vascular injury, blood clotting, systemic dysfunction

## Abstract

Sepsis poses a significant challenge due its lethality, involving multiple organ dysfunction and impaired immune responses. Among several factors affecting sepsis, monocytes play a crucial role; however, their phenotype, proteomic profile, and function in septic shock remain unclear. Our aim was to fully characterize the subpopulations and proteomic profiles of monocytes seen in septic shock cases and discuss their possible impact on the disease. Peripheral blood monocyte subpopulations were phenotype based on CD14/CD16 expression by flow cytometry, and proteins were extracted from the monocytes of individuals with septic shock and healthy controls to identify changes in the global protein expression in these cells. Analysis using 2D-nanoUPLC-UDMSE identified 67 differentially expressed proteins in shock patients compared to controls, in which 44 were upregulated and 23 downregulated. These proteins are involved in monocyte reprogramming, immune dysfunction, severe hypotension, hypo-responsiveness to vasoconstrictors, vasodilation, endothelial dysfunction, vascular injury, and blood clotting, elucidating the disease severity and therapeutic challenges of septic shock. This study identified critical biological targets in monocytes that could serve as potential biomarkers for the diagnosis, prognosis, and treatment of septic shock, providing new insights into the pathophysiology of the disease.

## 1. Introduction

Sepsis is a potentially fatal clinical syndrome characterized by organ dysfunction caused by the host’s deregulated response to infection [1]. This syndrome evolves into septic shock when the pathophysiological changes are intensified. This entails circulatory, cellular, and metabolic abnormalities becoming severe enough to cause disturbance, and is associated with an increased risk of death [2,3,4].

Arterial hypotension, a hallmark of septic shock, results from hemodynamic changes, including alterations in the degree of hypovolemic response, a decreased vascular tone, and myocardial depression [5]. One of the characteristics of this alteration is the increase in generalized vascular permeability, generating progressive subcutaneous and cavitary edema. This results in blood hypovolemia. Endothelial injury, causing vascular leakage and edema, may be responsible for shock, microvascular thrombosis, and organic failure [6,7]. In septic shock, systemic vascular dysfunction is observed as described above; however, these pivotal changes are essential to controlling the infection. The endothelial lesions trigger inflammatory activation and coagulation. Both are important to activating an immune response against the pathogens and to compartmentalizing the infection, preventing a systemic response. Subsequently, the rolling and adhesion of the white blood cells, and the increase in the vascular permeability, allow these cells to infiltrate tissues and combat invading microorganisms [3,8].

In this context, monocytes play an essential role since they are involved in systemic blood circulation. These cells are also part of the immune system’s first line of scouting defense, with the function of combating microorganisms, performing dead cell clearance, and undertaking injured tissue regeneration [9,10].

Monocytes are present in three distinct populations: classical (CD14high:CD16low), non-classical (CD14low:CD16high), and intermediate (CD14high:CD16high). Classical monocytes are pro-inflammatory, performing phagocytosis and releasing large amounts of reactive oxygen species. Non-classical monocytes are anti-inflammatory, primarily affecting tissue regeneration, immunological surveillance, and vascular endothelial patrol. Intermediate monocytes are monocytes that are transitioning from a classical to a non-classical profile and can display the characteristics of both profiles [11,12].

Understanding the complex biological and clinical roles of monocytes in septic shock remains a challenge. Many researchers have focused their findings and efforts on the cell response to microorganisms, including phagocytic capacity, microbicide, antigen presentation (HLA-DR), and cytokine production, in order to determine if the monocytes display any dysfunction during septic shock. Moreover, different monocyte expression patterns can be observed by examining a patient’s clinical history [10,13,14,15].

In the present study, we intended to evaluate the global protein profile from septic shock patients’ circulating monocytes in order to determine the existent alterations in these cells and to investigate their possible contribution to complete immune collapse and the subsequent mortality.

## 2. Results

### 2.1. Monocyte Profile

Following PBMC extraction, three monocyte populations were identified: CD14high:CD16low, CD14high:CD16high and CD14low:CD16high. Control subjects present about 50% CD14high:CD16low monocytes, 12% CD14high:CD16high monocytes, and 12% CD14low:CD16high monocytes. In septic shock patients, the CD14low:CD16high monocyte population was the most abundant in peripheral blood (approximately 70%); 2% of the cells were CD14high:CD16low, and 3% were CD14high:CD16low (Figure S1).

### 2.2. Mass Spectrometry and Bioinformatics

A total of 3472 proteins were identified in our study (1705 in control samples and 1727 in the septic shock samples), with an average of 7 peptides per protein. For the entire study, the coefficients of variance for all quantitative protein measurements were less than 6%. Sixty-seven proteins were differentially expressed in the monocytes from septic shock patients compared to controls. Considering at least 3 folds in the protein expression levels, 48 proteins were found to be increased, while 23 protein levels were decreased. The details of these proteins, including statistical values and cellular locations, are presented in Table 1.

**Figure 1:**
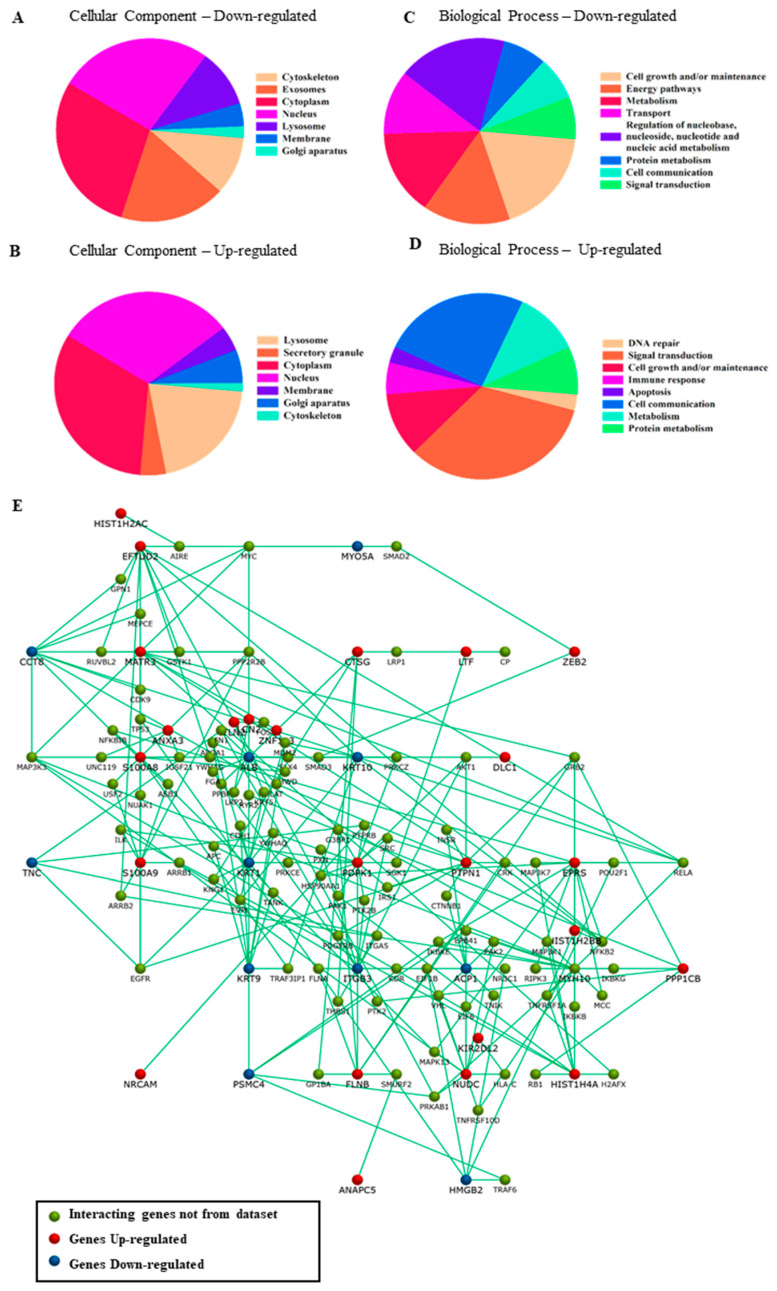
The biological components and interactions of differentially expressed proteins. The major cellular components involved are downregulated (**A**) and upregulated (**B**). The main biological processes participating in differentially expressed proteins are (**C**) down- and (**D**) upregulated. The interactions between differentially expressed proteins are not direct. However, they influence several molecules that communicate directly. Blue represents downregulated genes, red indicates upregulated genes, and green indicates the interaction genes of the FunRich database (**E**).

Gene ontology, FunRich, and Pathway Studio were used to analyze the changes present in the monocytes of septic shock patients. Major cellular components and biological processes involved in the altered proteins were identified using gene ontology analysis in FunRich (Figure 1).

Although differentially expressed proteins did not correlate directly with each other, they participated in common pathways, forming an interactome, with only one molecule required for communication between differentially expressed proteins (Figure 1). The Supplementary Table S2 describes the proteins that are interconnected.

The major regulatory molecules of proteins that are augmented are nuclear proteins with Enhancer-binding protein action, such as CEBPA, CEBPE, and CEBPP. The inflammasome and S100A3 protein in the cytoplasm and signaling pathways are activated by HIF-1a, interleukin 17, and lactoferrin (Table 2).

Proteins that have decreased expressions are mainly influenced by transcriptional factors GRHL2 and NFX1; growth factors TGFA and FGF2; integrins ITGA3 and ILK; and PLAT and Oncostatin M proteins, with actions described in Table 3.

To assess whether these protein changes influence monocyte function, we examined their effects on phenotype, cellular activation, phagocytosis, migration, senescence, cell viability, and apoptosis/necrosis.

Among the 67 differentially expressed proteins, 46 (29 upregulated and 17 downregulated) were associated with these functions, but it remains unclear which changes are most relevant for monocyte dysfunction (Table S3).

Resistin, S100A8, S100A9, MPO, LCN2, PDPK1, CSTG, LTF, PTP1, and EPRS are proteins that may act directly in the collapse of sepsis since they are increased in septic shock cases and can be secreted by monocytes. These proteins have been correlated with direct effects in situations of endotoxemia, chronic inflammation, vascular damage, and thrombosis (Table 4).

## 3. Discussion

Monocytes play a crucial role in orchestrating the host immune response and contribute to the pathogenesis of sepsis. In the early stages of this syndrome, monocytes exacerbate the immune response through a cytokine storm. As the disease progresses, they contribute to immunological exhaustion and adopt an immunosuppressive phenotype, being incapable of responding to secondary infections [103,104,105].

Classical, intermediate, and non-classical monocytes have distinct predominant functions [106]. Disturbance in the proportion of these monocytes subsets in the blood has been linked to several serious pathologies with poor clinical outcomes [107,108,109].

In this study, patients with septic shock exhibited a predominance of non-classical monocytes in circulation. Non-classical monocytes play a crucial role in septic shock by serving as potential diagnostic markers and indicators of disease severity. Research has shown that the proportion of non-classical monocytes is significantly higher in septic patients compared to non-septic individuals, indicating their potential value in diagnosing sepsis [110]. Additionally, the frequency of classical and intermediate monocytes at the time of ICU admission can predict short-term survival in patients with septic shock, with distinct differences observed between survivors and non-survivors [107]. Furthermore, the dynamic nature of sepsis involves a shift in monocyte polarization, with an increase in intermediate monocytes in patients with suspected or confirmed sepsis, highlighting their role in the immune response during disease progression [111]. Additionally, an increase in non-classical monocytes in patients with clinical symptoms of sepsis was reported to be associated with negative bacterial blood cultures, while an increase in intermediate monocytes population was associated with positive blood cultures [112,113], suggesting that non-classical monocytes may play a role in controlling intermediate infection disruptions. These findings underscore the significance of non-classical monocytes in septic shock, and as potential diagnostic and prognostic markers.

Patients with high proportions of non-classical classical monocytes and low proportions of classical monocytes exhibit impaired endothelial function; as such, it is recognized that high levels of non-classical monocytes are associated with increased vascular superoxide production, leading to vascular dysfunction [11,114,115].

Although immunophenotyping indicated the predominance of non-classical monocytes in septic shock patients, the S100A9, CTSG, MPO and RNAS3 proteins, which are upregulated, have been identified in intermediate and classical monocytes [11,116,117]. The large increase in non-classical monocytes in the circulation of the analyzed patients may be the result of rapid migration without complete differentiation.

As noted, there is an apparent paradox between the predominance of non-classical monocytes, which are typically associated with anti-inflammatory and tissue repair functions in healthy individuals, and the pro-inflammatory and prothrombotic protein expression observed in our septic shock cohort. This shift in phenotype could be explained by the extreme inflammatory environment of septic shock, which may induce atypical activation states in these cells. The rapid recruitment and incomplete differentiation of non-classical monocytes may drive them into a more pro-inflammatory role, as evidenced by the upregulation of proteins like S100A9, CTSG, and MPO. This atypical behavior mirrors the findings of [118], who described monocytes with M2-like features as exhibiting inflammatory activity in septic patients. Such a shift suggests a maladaptive immune response, where non-classical monocytes contribute to immune dysregulation under the severe conditions of septic shock.

The Enhancer-binding protein, HIF-1a, and the inflammasome are major regulatory molecules in the transcription of various genes, culminating in increased expression of different proteins. They play key roles in the regulation of monocytes. (Table 2).

The Enhancer-binding protein regulates almost all listed proteins. In addition, it has been associated with essential proteins for the differentiation of classical monocytes into non-classical ones [119,120,121]. On the other hand, hypoxia-inducible factor-1α (HIF-1α) induces immunosuppression and enhances repair characteristics in classical and intermediate monocytes during active sepsis. It regulates metabolic pathways, promotes glycolysis, and reduces oxidative phosphorylation, adapting monocyte metabolism to hypoxic conditions. HIF-1α also supports cellular survival, repair, and differentiation into anti-inflammatory subtypes, while enhancing phagocytic activity in order to effectively clear pathogens [122,123].

The inflammasome, a crowded multiprotein complex, finely regulates caspase-1 to induce pro-inflammatory protein expression. During septic shock, the activity of the inflammasome, particularly the NLRP3 inflammasome, is dysregulated, which contributes to immune system imbalance and monocyte deactivation [124,125,126,127].

Many studies have identified monocyte dysfunction in septic shock cases, particularly regarding changes in their ability to present antigens and produce cytokines. However, it is known that their phagocytic and microbicidal functions remain conserved [128,129].

In response to pathogens, monocytes can secrete molecules that have a direct effect on the collapse of septic shock (Table 4).

Defense mechanisms are beneficial when activated locally, but harmful when systemically activated. Septic shock results from generalized consequences of the immune response to infection, which involves a cascade of events including endotoxemia, inflammation, vascular damage, and thrombosis [130,131].

Endotoxemia is correlated with infection per se. It involves components of the pathogen’s cell wall being present in abundance in the systemic circulation. While Gram-negative bacteria are frequently associated with endotoxemia, high levels of endotoxin have also been found in the plasma of patients with septic shock caused by Gram-positive bacteria, fungi and, in some cases, viruses [132,133,134]. 

The presence of endotoxins activates the immune system cells. This process leads to utter inflammation and disturbance in the production of pro-inflammatory cytokines, such as TNF-α, IL-6, IL-1β, IL-8, and IL-12, as well as in the production of platelet activation factors, reactive oxygen species, and microbicidal components (myeloperoxidase, cationic proteins, acid hydrolases, cathepsin G, and lactoferrin), which struggle against microorganisms [135,136,137,138]. However, the exacerbation of these factors causes damage to the vascular endothelium [6,7,8].

Prolonged inflammation and the presence of endothelial injury push homeostasis toward a prothrombotic and anti-fibrinolytic state, which can lead to disseminated microvascular thrombosis, organ ischemia, and multiple organ dysfunction syndrome, common and well-known circumstances that follow septic shock [139,140].

## 4. Materials and Methods

### 4.1. Study Population

The Ethics Committee of the Federal University of Uberlândia (UFU) approved this study under protocol number 153.331. Prior to blood collection, written informed consent was obtained from all controls and patients or their guardians.

#### 4.1.1. Healthy Volunteers

The control group consisted of 10 healthy individuals, including 5 women and 5 men (age range, 19–55 years; median age, 27 years). Exclusion criteria included HIV infection, autoimmune disease, or any condition compromising full health.

#### 4.1.2. Patients

Five patients, who were in treatment at the Intensive Care Unit in the Clinical Hospital—Federal University of Uberlândia (Uberlândia—Brazil), were included in this study. All patients were over than 18 years and fulfilled the defining criteria of septic shock [4]. Samples were collected within 48 h of diagnosis. Relevant clinical and laboratory data for septic shock patients are displayed in Supplementary Table S1.

### 4.2. Peripheral Blood Monocytes Isolation

Approximately 40 mL of blood was collected from patients and control subjects in tubes containing Heparin (an anti-coagulant). Fresh blood samples were immediately processed to isolate peripheral blood mononuclear cells (PBMCs) using density gradient centrifugation through Histopaque-1077 (Sigma, Sigma, St. Louis, MO, USA, Catalog n° H1077-1). Subsequently, monocytes were isolated from PBMCs using the Dynabeads untouched human monocytes kit, (Thermo Fisher Scientific, Waltham, MA, USA, Catalog n°11350D) according to the manufacturer’s instructions. The kit contains antibodies and magnetic beads to capture T cells, B cells, NK cells, dendritic cells, erythrocytes, granulocytes, and macrophages, leaving monocytes untouched and free of surface-bound antibodies and beads. Monocyte populations in PBMCs were characterized by flow cytometry using CD14 and CD16 antibodies (Bio-legend: San Diego, CA, USA: CD14 PECy7, catalog: 325618/400126, and CD16 Alexa Fluor 647, catalog: 302020/400130).

### 4.3. Protein Extraction and Sample Preparation

Monocytes total proteins were extracted using Complete Lysis-M, EDTA-free (Sigma Aldrich, St. Louis, MO, USA, catalog: 04719964001), according to the manufacturer’s protocol. Proteins were concentrated using Amicon ultra-filtration devices with 10 kDa molecular weight cutoff membranes (Merck-Millipore, Darmstadt, Germany, catalog: UFC501096) and quantified by the Micro BCA Protein assay kit (Thermo Fisher Scientific, Waltham, MA, USA, catalog: 23235). Samples were adjusted to the same protein concentration and digested with trypsin, as described below. Each pooled sample (50 μg of total protein) was buffer-exchanged in 50 mM ammonium bicarbonate, denatured in the presence of 0.2% RapiGEST SF (Waters, Milford, MA, USA) at 80 °C for 15 min in a dry bath, reduced with 100 mM dithiothreitol at 60 °C for 60 min, and then alkylated with 300 mM iodoacetamide for 30 min in the dark at room temperature. Samples were then digested with modified trypsin (Promega, Madison, WI, USA) at an enzyme-to-protein ratio of 1:100 (*w*/*w*) at 37 °C overnight. The reaction was stopped using 10 μL of 5% (*v*/*v*) trifluoroacetic acid (TFA), mixed, and incubated for 90 min at 37 °C. Samples were centrifuged at 14,000 rpm at 4 °C for 30 min (10). The supernatant was recovered and then reconstituted in ammonium hydroxide (NH_4_OH) previously prepared at 1 N and then transferred to a Waters Total Recovery vial (Waters) prior to 2D-nanoUPLC-UDMSE analyses. Tryptic digested peptides from yeast alcohol dehydrogenase (ADH) (Waters, Milford, MA, USA) were added to a final concentration of 25 fmol.μL^−1^ as an internal standard for relative quantification and column loading capacity estimation. The quantitative method utilized in the above estimation is described in detail by Jeffrey Silva et al. (2006) and was performed accordingly [141]. Each sample were injected during “scouting” runs for stoichiometry purposes between assessments of the conditions using an integrated total ion current, as previously described [142].

### 4.4. Mass Spectrometry of Complex Digested Samples

Using a 2D-nanoUPLC-tandem nanoESI-UDMSE instrument platform, proteomic analyses were performed through multiplexed data-independent acquisition experiments [143].

Samples were fractionated using a dual reversed-phase (RP) approach. In first-dimension chromatography, peptides (5 µg) were loaded onto an M-Class BEH C18 Column (130 Å, 5 µm, 300 µm × 50 mm, Waters Corporation, Milford, MA, USA). Fractionation was performed through 10 discontinuous steps involving acetonitrile (8.7%, 11.4%, 13.2%, 14.7%, 16.0%, 17.4%, 18.9%, 20.7%, 23.4%, 50%) and via high-pH fractionation over 10 min at a flow rate of 2 µL.min^−1^. After each step, peptide loads were carried through to second-dimension separation on a nanoACQUITY UPLC HSS T3 Column (1.8 µm, 75 µm × 150 mm, Waters Corporation). 

Peptide elution was performed using a continuous acetonitrile gradient from 7% to 40% (*v*/*v*) over 54 min at a flow rate of 0.4 µL.min^−1^ directly into a Synapt G2-S HDMS. For every measurement, the mass spectrometer was operated in resolution mode with the scan time (0.5 s) adjusted to obtain at least 10 points per chromatographic peak for each low and elevated energy level (σ10%:20). The *m*/*z* resolving power was approximately 1,800,000 FWHM when considering a conjoined stacked-ring ion method such as the T-wave ion mobility, which operates with a cross-section resolving power of at least 40 Ω/ΔΩ. LC–MS/MS multiplex data were collected using ion-mobility-enhanced MSE [144,145].

The exact mass retention time (EMRT) signals from multiplexed ion-mobility DIA scanning (UDMSE) were detected in an alternating low-energy and elevated-energy acquisition mode. In the low-energy mode, data were collected at 6 eV. In the elevated collision energy, quasi *m*/*z*-specific collision energies were applied at the traveling-wave stacked-ring ion guide transfer lens (TWIG) to the different drift time bins [146]. These were used to fragment precursor ions prior to orthogonal acceleration time-of-flight (oaTOF) analysis, at the transfer TWIG cell filled with argon gas, using collision-induced dissociation (CID) [147].

The mass spectrometer was calibrated with an MS/MS spectrum of human [Glu1]-Fibrinopeptide B (Glu-Fib) that was delivered every 30 s through the reference sprayer of the NanoLock Spray source. Quadrupole profiles were adjusted if an *m*/*z* less than 400 arose from dissociations in the collision cell. Global quality control of the obtained data and the figures of merit (FOMs) are displayed as described at Souza et al., 2017 (Figure S2) [147].

### 4.5. Database Searching and Bioinformatics

Proteins were identified and quantified using dedicated algorithms and searched against the UniProt human proteomic database, version 2016_02 [147,148]. For proper spectra processing and database searching conditions, Progenesis QI V3.0 for Proteomics software with Apex3D, Peptide 3D, and Ion Accounting informatics were exploited (Waters Corporation). Processing parameters included 150 counts for the low-energy threshold, 50.0 counts for the elevated energy threshold, and 750 counts for the intensity threshold as a default, such as peak picking and alignment processing [149]. The following parameters were considered for protein identification and quantitation: (1) digestion by trypsin with at least one missed cleavage; (2) variable modifications by oxidation (M) and fixed modification by carbamidomethyl (C); and a (3) false discovery rate (FDR) of less than 1%. We used at least three peptides per protein, with one unique aspect, and performed quantitation based on non-conflicted peptides per condition. Only reproducible proteins were investigated across all the replicates obtained. Identifications and quantitative values that did not satisfy these criteria were rejected. The raw files and the database search tables were deposited in the ProteomeXchange repository under accession number PXD004696.

#### Functional Annotation and In Silico Analysis of Monocyte Function

Functional annotations (gene ontology and chromosome annotations) of the dataset were performed using DAVID Bioinformatics Resources 6.8 (https://david.ncifcrf.gov/home.jsp (accessed on 4 March 2017)). Gene ontology (GO) functional classifications were analyzed with Blast2GO software V6.0.1 (www.blast2go.org (accessed on 6 April 2017)) and GO enrichment analysis was conducted to identify GO terms that were significantly enriched in differentially expressed proteins. We also utilized FUNRICH software V3.1.4 (http://www.funrich.org/ (accessed on 18 May 2017)) to evaluate the interactome and assess the functional enrichment of pathways associated with monocyte activities.

In silico analysis was performed to predict the potential impact of the identified protein changes on key monocyte functions, including phenotype, cellular activation, phagocytosis, migration, senescence, cell viability, and apoptosis/necrosis. Using gene ontology, we categorized the proteins based on their associated biological processes, molecular functions, and cellular components, which are linked to monocyte behavior. FunRich was employed to identify enriched pathways and potential protein interactions relevant to monocyte activation and migration. Furthermore, Pathway Studio Software V21.0 (www.pathwaystudio.com (accessed on 22 June 2017)) was used to explore regulatory networks and signaling pathways, allowing us to predict the influence of the identified proteins on processes such as cellular activation, apoptosis, and necrosis. These tools enabled us to model how protein alterations observed in monocytes during septic shock might affect their function.

## 5. Study Limitations

This study has limitations, such as the small sample size, and the confirmation regarding the impact of global proteome alterations in monocytes being based solely on in silico analyses and supported only by a literature review. However, this is the first study to demonstrate how monocytes can modify the microenvironment and contribute to the immunopathological collapse in septic shock, providing new insights into the role of these cells in the progression of the disease.

## 6. Conclusions

Sepsis is a syndrome in which the human system undergoes extreme immune and pathophysiological stress, characterized by initial immune system exacerbation and later physiological response silencing. Monocyte changes in sepsis shock suggest a “biological reprogramming” rather than a simple loss of function. In this study, we presented robust evidence on how the monocyte systemic effect occurs upon the septic shock collapse. Despite limitations, such as pool samples and the absence of separation of the monocytes in subtypes, we demonstrated the potential contribution of monocytes to the collapse of the septic shock. Further studies are needed to validate these findings and to ascertain the contribution of each individual protein in monocyte functional alterations during sepsis. However, these findings could have important clinical implications. The observed monocytes alterations may serve to evaluate the progression in septic shock. By addressing immune dysfunction and vascular damage, these insights may contribute to the future development of personalized treatment strategies, potentially improving patient outcomes and reducing mortality rates.

## Figures and Tables

**Table 1:** Differentially expressed proteins identified in the monocytes of patients with septic shock compared with healthy controls.

Gene	Accession	Description	Score	ANOVA (*p*)	Ratio	N ^1^	C ^1^	M ^1^	S ^1^	R ^2^
ANXA3	P12429	Annexin A3	171.36	6.04 × 10^−6^	3.046					+
HIST1H2AC	Q93077	Histone H2A type 1-C	44.50	7.00 × 10^−6^	3.087					+
FLNB	O75369	Filamin-B	155.76	3.045 × 10^−8^	3.121					+
GXYLT2	A0PJZ3	Glucoside xylosyltransferase 2	9.12	6.82 × 10^−5^	3.122					+
IGSF10	Q6WRI0	Immunoglobulin superfamily member 10	63.37	2.88 × 10^−6^	3.125					+
WDR90	Q96KV7	WD repeat-containing protein 90	46.32	0.0002	3.146					+
ZEB2	O60315	Zinc finger E-box-binding homeobox 2	7.80	0.0001	3.167					+
EPRS	P07814	Bifunctional glutamate/proline--tRNA ligase	19.40	9.10 × 10^−5^	3.168					+
HYDIN	Q4G0P3	Hydrocephalus-inducing protein homolog	91.08	5.43 × 10^−6^	3.187					+
ADGRD1	Q6QNK2	Adhesion G-protein coupled receptor D1	13.29	5.89 × 10^−6^	3.239					+
RETN	Q9HD89	Resistin	24.98	0.0001	3.251					+
LCN2	P80188	Neutrophil gelatinase-associated lipocalin	53.16	1.11 × 10^−6^	3.255					+
PDPK1	O15530	3-phosphoinositide-dependent protein kinase 1	15.67	0.0001	3.284					+
NNMT	P40261	Nicotinamide N-methyltransferase	13.64	1.56 × 10^−5^	3.286					+
HIST1H2BK	O60814	Histone H2B type 1-K	68.54	5.53 × 10^−5^	3.356					+
RNASE3	P12724	Eosinophil cationic protein	35.48	0.0002	3.359					+
ATP6V1B1	C9JL73	V-type proton ATPase subunit B, kidney isoform	45.81	2.28 × 10^−6^	3.436					+
DLC1	Q96QB1	Rho GTPase-activating protein 7	10.11	3.05 × 10^−6^	3.452					+
HIST1H2BB	P33778	Histone H2B type 1-B	75.93	6.93 × 10^−6^	3.540					+
MLH3	Q9UHC1	DNA mismatch repair protein Mlh3	36.53	4.81 × 10^−7^	3.599					+
ZNF133	P52736	Zinc finger protein 133	7.84	0.0001	3.603					+
SACM1L	Q9NTJ5	Phosphatidylinositide phosphatase SAC1	25.19	6.30 × 10^−6^	3.625					+
TLN2	Q9Y4G6	Talin-2	139.60	1.56 × 10^−5^	3.833					+
LTF	P02788	Lactotransferrin	207.35	5.46 × 10^−6^	4.066					+
PPP1CB	P62140	Serine/threonine–protein phosphatase PP1-beta catalytic subunit	36.26	1.12 × 10^−5^	4.134					+
S100A8	P05109	Protein S100-A8	68.05	3.52 × 10^−5^	4.458					+
PTPN1	B4DSN5	Tyrosine–protein phosphatase non-receptor type	25.30	0.0001	4.506					+
KLB	Q86Z14	Beta-klotho	15.35	6.19 × 10^−6^	4.515					+
ANAPC5	F5H0F9	Anaphase-promoting complex subunit 5	14.87	7.71 × 10^−5^	4.525					+
CTSG	P08311	Cathepsin G	137.54	2.36 × 10^−7^	4.539					+
CHTF8	P0CG12	Chromosome transmission fidelity protein 8 homolog isoform 2	23.38	1.11 × 10^−5^	5.026					+
C8B	P07358	Complement component C8 beta	13.87	1.36 × 10^−6^	5.059					+
MPO	P05164	Myeloperoxidase	350.67	1.19 × 10^−6^	5.399					+
EFTUD2	Q15029	116 kDa U5 small nuclear ribonucleoprotein component	51.23	3.93 × 10^−6^	5.491					+
	K7ERQ8	Uncharacterized protein (Fragment)	18.18	1.15 × 10^−5^	5.579					+
TUBGCP5	Q96RT8	Gamma-tubulin complex component	9.59	4.57 × 10^−5^	5.580					+
FAM167A	Q96KS9	Protein FAM167A	15.10	2.78 × 10^−6^	6.212					+
NRCAM	C9JYY6	Neuronal cell adhesion molecule	8.18	2.43 × 10^−5^	6.232					+
S100A9	P06702	Protein S100-A9	141.73	3.65 × 10^−6^	7.217					+
MATR3	P43243	Matrin-3	31.47	6.77 × 10^−6^	7.618					+
HIST1H4A	P62805	Histone H4	132.30	2.00 × 10^−6^	10.050					+
CEACAM6	P40199	Carcinoembryonic antigen-related cell adhesion molecule 6	28.45	1.75 × 10^−5^	11.350					+
KIR2DL2	Q8N742	KIR2DL2	14.60	1.18 × 10^−5^	11.410					+
NUDC	Q9Y266	Nuclear migration protein nudC	15.40	3.04 × 10^−76^	11.518					+
SH3TC2	E9PDF1	SH3 domain and tetratricopeptide repeat-containing protein 2	28.98	2.27 × 10^−6^	18.110					−
KRT9	P35527	Keratin, type I cytoskeletal 9	10.62	1.42 × 10^−7^	9.427					−
ACP1	P24666	Low-molecular-weight phosphotyrosine protein phosphatase	9.94	1.08 × 10^−5^	5.852					−
KRT1	P04264	Keratin, type II cytoskeletal 1	57.80	6.76 × 10^−7^	5.782					−
KRT10	P13645	Keratin, type I cytoskeletal 10	137.21	6.77 × 10^−6^	5.695					−
LZTR1	Q8N653	Leucine-zipper-like transcriptional regulator 1	11.91	4.68 × 10^−6^	4.618					−
ATP13A3	Q9H7F0	Probable cation-transporting ATPase 13A3	28.15	1.61 × 10^−5^	4.614					−
ZNF677	Q86XU0	Zinc finger protein 677	7.78	4.14 × 10^−5^	4.543					−
ITGB3	P05106	Integrin beta-3	174.14	2.37 × 10^−6^	4.476					−
TNC	P24821	Tenascin	36.37	8.73 × 10^−6^	4.307					−
ZFHX2	Q9C0A1	Zinc finger homeobox protein 2	14.11	0.0001	4.104					−
ESRP2	Q9H6T0	Epithelial splicing regulatory protein 2	16.19	1.7 × 10^−5^	4.011					−
MYH10	P35580	Myosin-10	76.79	3.25 × 10^−5^	3.879					−
COG4	Q9H9E3	Conserved oligomeric Golgi complex subunit 4	14.50	0.0001	3.870					−
PSMC4	P43686	26S protease regulatory subunit 6B	10.90	8.76 × 10^−5^	3.762					−
ATAD2B	Q9ULI0	ATPase family AAA domain-containing protein 2B	13.66	8.72 × 10^−5^	3.703					−
PGK2	P07205	Phosphoglycerate kinase 2	94.21	2.49 × 10^−6^	3.701					−
ALB	P02768	Serum albumin	164.05	4.34 × 10^−5^	3.670					−
ACAD9	Q9H845	Acyl-CoA dehydrogenase family member 9, mitochondrial	14.60	4.86 × 10^−6^	3.262					−
CCT8	P50990	T-complex protein 1 subunit theta	76.19	8.92 × 10^−6^	3.255					−
HMGB2	P26583	High-mobility group protein B2	63.46	2.43 × 10^−5^	3.059					−
CLCN4	P51793	H(+)/Cl(−) exchange transporter 4	8.30	0.0003	2.927					−
MYO5	G3V394	Unconventional myosin-Va	128.70	1.25 × 10^−6^	2.855					−

^1^ Black squares indicate cellular location: N (nucleus); M (membrane); C (cytoplasm); S (secreted); ^2^ protein regulation: + (upregulation); − (downregulation).

**Table 2:** The primary regulation of proteins with increased expression in septic shock.

Protein	Effector	Effect	Ref.
LTF	CEBPE	CEBPE is involved in the positive regulation of lactoferrin gene expression	[16]
	CEBPA	C/EBPα binds to the C/EBP site in the lactoferrin promoter in induced myeloid cells	[17]
MPO	CEBPE	Upregulation of CBPE induces expression of myeloperoxidase	[18]
	LTF	LTF decreased the serum C-reactive protein level and the inducible nitric oxide synthase (iNOS) and myeloperoxidase (MPO) gene expression levels	[19]
	HIF-1	Activation of HIF-1 by P4HA2 gene silencing attenuated myeloperoxidase expression in myocardium following ischemia–reperfusion	[20]
	IL1-RA	Interleukin-1ra and anti-TNF-α also significantly lowered MPO levels	[21]
	CEBPA	C/EBPα inhibits monocyte/macrophage differentiation and initiates granulocyte differentiation by inducing myeloperoxidase gene expressions	[22]
RETN	CEBPE	Human leukocyte resistin expression depends on the myeloid-specific nuclear transcription factor CEBPE	[23]
	CEBPB	Resistin stimulates the expression of chemokine genes in chondrocytes via the combinatorial regulation of C/EBPβ and NF-κB.	[24]
	HIF-1	Hypoxia-inducible factor-1 results in the increased production of leptin, resistin, TNF, and IL-6	[25]
	IL1-RA	IL-1Ra treatment for reduction in leptin and resistin levels	[26]
	CEBPA	Resistin gene promoter carries the C/EBP-α binding site, which is necessary and sufficient for transcription from the resistin gene promoter	[27]
LCN2	CEBPE	Stable inducible expression of CEBPE in the murine fibroblast cell line NIH 3T-activated expression of mRNA LCN2	[28]
	INFLAMMASOME	Inflammasome-mediated production of antimicrobial peptides, including Reg3β, Reg3γ, S100A8, S100A9, and LCN2	[29]
	CEBPB	LCN2 promoter region contains the binding sites of several transcription factors such as NF-κB, STAT1, STAT3, and CEBPB	[30]
	IL17	IL17 positively regulates LCN2 expression.	[31]
	CEBPA	IL6 and LCN2 promoters require both NF-κB and C/EBP elements	[32]
S100A8S100A9	S100A3	S100A3 co-expression inhibits AP-1 and NF-κB-dependent transcription upon S100A8 and S100A9 over-expression	[33]
	INFLAMMASOME	Inflammasome-mediated production of antimicrobial peptides, including Reg3β, Reg3γ, S100A8, S100A9, and LCN4	[29]
	LTF	Lactoferrin induces the production of chemokines (MIP-1a, MCP-1, and S100A9)	[34]
	CEBPB	IL-1α-induced S100A9 expression is signaled through the IL-1 receptor and p38 MAPK pathways, resulting in the binding of CEBPB to the upstream S100A9 promoter	[35,36]
	HIF-1	Hypoxia and HIF-1 increase S100A8 and S100A9 expression	[37]
	IL1-RA	IL-1Ra inhibited interleukin-1 α-induced S100A8 and S100A9 gene expression	[38]
	IL17F	IL-17 stimulates the expression of human beta-defensin- 2, S100A9 and enhances the expression of S100A7 and S100A9	[39]
	CEBPA	Increase in the presence of reactive oxygen species and the expression levels of the transcription factors Klf-5 and CEBPA in neutrophils, both of which promote S100A8/S100A9 expression	[40]
PDPK1	LTF	Lactotransferrin downregulates the level of 3-phosphoinositide-dependent protein kinase 1 (PDK1) transcription and subsequently inhibits other proteins	[41]
	HIF-1	Repression of hypoxia-inducible factor-1 activity, attenuate PDK-1 expression	[42]
HIST4H4	CEBPB	CEBPB can significantly transactivate the expression of HIST4H4	[43]
NNMT	CEBPB	NNMT promoter region also contains the consensus sequences for signal transducers and activators of transcription binding elements	[44]
CEACAM6	HIF-1	Hypoxia-inducible factor-1 transcription factor increases CEACAM6 expression in intestinal epithelial cells	[45]

**Table 3:** The main regulation of proteins with low expression in septic shock.

Protein	Effector	Function Protein Effector	Effect	Ref.
KRT1	GRHL2	Transcription factor	Downregulate KRT1 and KRT10	[46]
	NFX1	Nuclear transcription factor	Upregulate KRT1 and KRT10	[47]
	BCR	Breakpoint cluster region protein	Loss of BCR reduces expression of KRT1 and KRT10	[48]
	PLAT	Plasminogen activator, tissue	Plays a role in the expression of KRT1 and KRT10	[49]
	OSM	Oncostatin M	Downregulate mRNA expression	[50]
KRT10	GRHL2	Transcription factor	Downregulate KRT1 and KRT10	[46]
	NFX1	Nuclear transcription factor	Upregulate KRT1 and KRT10	[47]
	COLLAGEN	Matrix protein	Downregulate the mRNA expression KRT10	[51]
	BCR	Breakpoint cluster region protein	Loss of BCR reduces expression of KRT1 and KRT10	[48]
	TGFA	Transforming grown factor	Suppress KRT10 expression, promoted late terminal differentiation	[52]
	FGF2	Fibroblast growth factor 2	Downregulate the KRT10 expression	[53]
	PLAT	Plasminogen activator, tissue	Plays a role in the expression of KRT1 and KRT10	[49]
	ITGA3	Integrin alpha 3	Inhibition KRT10 production	[54]
	OSM	Oncostatin M	Decrease expression	[55]
ABL	GRHL2	Transcription factor	Inhibits expression ABL	[56]
	COLLAGEN	Matrix protein	Inhibit albumin production at short times, but enhances albumin production at longer times	[57]
	TGFA	Transforming grown factor	Stimulate albumin synthesis	[58]
	FGF2	Fibroblast growth factor 2	Induce expression	[59]
	ILK	Integrin like kinase	ALB mRNA expression is downregulated by ILK	[60]
	OSM	Oncostatin M	Upregulate production	[61]
ITGB3	COLLAGEN	Matrix protein	Enhance β3 integrin tyrosine phosphorylation by adhesion platelets to collagen	[62]
	TGFA	Transforming grown factor	Stimulate ITGB3 expression	[63]
	ITGA3	Integrin alpha 3	Enhance the expression	[64]
TNC	COLLAGEN	Matrix protein	Tenascin-C mRNA expression is reduced by native collagen and is upregulated by denatured collagen	[65]
	FGF2	Fibroblast growth factor 2	Upregulator of tenascin expression and activation	[66]
	ILK	Integrin like kinase	Induce expression TNC	[67]
	OSM	Oncostatin M	Inhibit mRNA expression	[68]
MYH10	FGF2	Fibroblast growth factor 2	Decrease MYH10 expression	[69]
	ILK	Integrin like kinase	Regulate MYH10 expression	[70]
ESRP2	FGF2	Fibroblast growth factor 2	Repress the levels of ESRP2 mRNA	[71]
PGK2	FGF2	Fibroblast growth factor 2	Modulate transcription of PGK-2 genes	[72]

**Table 4:** Systemic effect of secreted proteins upregulation on septic shock.

Protein	Effect	Action	Ref.
RETN	Inflammation	Mediated chronic inflammation can lead to atherosclerosis, and other cardiometabolic diseases	[73]
	Vascular damage	Major inducer of endothelial damage through the induction of permeability	[74]
	Thrombosis	Resistin an adipokine associated with the metabolic syndrome is believed to have a role in thrombotic conditions.	[75]
S100A8	Endotoxemia	S100A8 administration attenuated inflammation and injury in a mouse model of endotoxemia	[76]
	Inflammation	S100A8 important mediators of various processes during chronic inflammation	[77]
	Vascular damage	High S100A8 expression leads to endothelial damage by inducing the apoptosis and death of endothelial cells	[78]
	Thrombosis	S100A8 which is secreted by trophoblast cells probably regulates the level of macrophage activation and procoagulant factors	[79]
S100A9	Inflammation	S100A9, an important pro-inflammatory mediator in acute and chronic inflammation	[80]
	Vascular damage	S100A8/S100A9 is released in high amounts at sites of inflammation, S100A8/S100A9-induced endothelial damage	[81]
	Thrombosis	Platelet-derived S100 family member myeloid-related protein-14 regulates thrombosis	[82]
MPO	Endotoxemia	MPO can aggravate this inflammatory response in rodent models of endotoxemia	[83]
	Inflammation	MPO plays an important role in the initiation and progression of acute and chronic inflammation.	[84]
	Vascular damage	MPO consumes nitric oxide, leading to vasoconstriction and promoting endothelial damage	[85]
	Thrombosis	Hemoglobin-Hp2-2 complexes may promote a pro-inflammatory macrophage phenotype via oxidative mechanisms (MPO) leading to plaque destabilization and atherothrombosis	[86]
LCN2	Endotoxemia	Acute endotoxemia is associated with upregulation of lipocalin 24p3/Lcn2 in lung and liver	[87]
	Inflammation	LCN2 is involved in chronic inflammation	[88]
	Vascular damage	LCN2 supposedly mediates vascular damage and plaque rupture.	[89]
	Thrombosis	LCN2 could have an important role in thrombotic events associated with polycythemia vera and essential thrombocythemia	[90]
PDPK1	Inflammation	Deletion of PDPK1 induces chronic inflammation of the intestine	[91]
	Thrombosis	PDK1 is required for Ca(2+)-dependent platelet activation on stimulation of collagen receptor glycoprotein VI, arterial thrombotic occlusion, and ischemic stroke in vivo	[92]
CTSG	Inflammation	CTSG is thought to contribute to self-propagating, chronic inflammation.	[93]
	Vascular damage	CTSG causes the activation of bystander platelets, enhances vascular damage and inhibits fibrinolysis	[94]
	Thrombosis	CTSG is a potent platelet activator and promotes intravascular thrombosis, thus contributing to the formation of a thrombus clot	[95]
LTF	Endotoxemia	Lactoferrin suppresses endotoxemia by interfering with lipopolysaccharide dependent TLR4 activation	[96]
	Inflammation	Persistent production of lactoferrin in pediatric patients may contribute to chronic inflammation in the rectum	[97]
	Thrombosis	Lactoferrin may play a role in the pathogenesis of disseminated intravascular coagulation and thrombotic complications	[98]
PTPN1	Endotoxemia	PTP1 not protect lipopolysaccharide-induced inflammation, hyperinsulinemia, and endotoxemia through an IL-10 STAT3-dependent mechanism.	[99]
	Vascular damage	Cytokines, ROS, and COX trigger an acute inflammatory response and induce vascular damage that may be reduced by PTP1B deletion	[100]
EPRS	Endotoxemia	EPRS inhibition has beneficial effects against organ dysfunction due to reperfusion injury and endotoxemia	[101]
	Vascular damage	PARS activation plays a role in the pathogenesis of endothelial injury in endotoxin shock.	[102]

## Data Availability

The original contributions presented in this study are included in this article/Supplementary Material; further inquiries can be directed to the corresponding author.

## Supplementary Materials

The supporting information can be downloaded at: https://www.mdpi.com/article/10.3390/ijms251910641/s1.
