# Supplementary material for: The Modulation of Septic Shock: A Proteomic Approach
Source: Int J Mol Sci. 2024 Oct 3;25(19):10641. doi: 10.3390/ijms251910641 (PMC11476436; doi:10.3390/ijms251910641)
Supplement: Supplementary file 1 [file ijms-25-10641-s001.zip › ijms-3203907-supplementary.pdf]

International Journal of
Molecular Sciences

MDPI

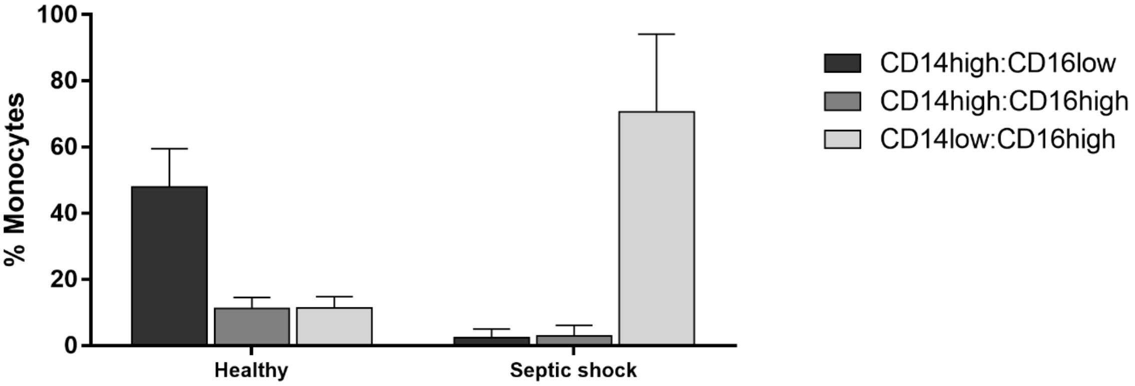

Figure S1: Monocyte's profile. Percentage of classical (CD14high:CD16low), intermediate (CD14high:CD16high), and non-classical (CD14low:CD16high) monocytes expression in PBMC from patients and controls evaluated.

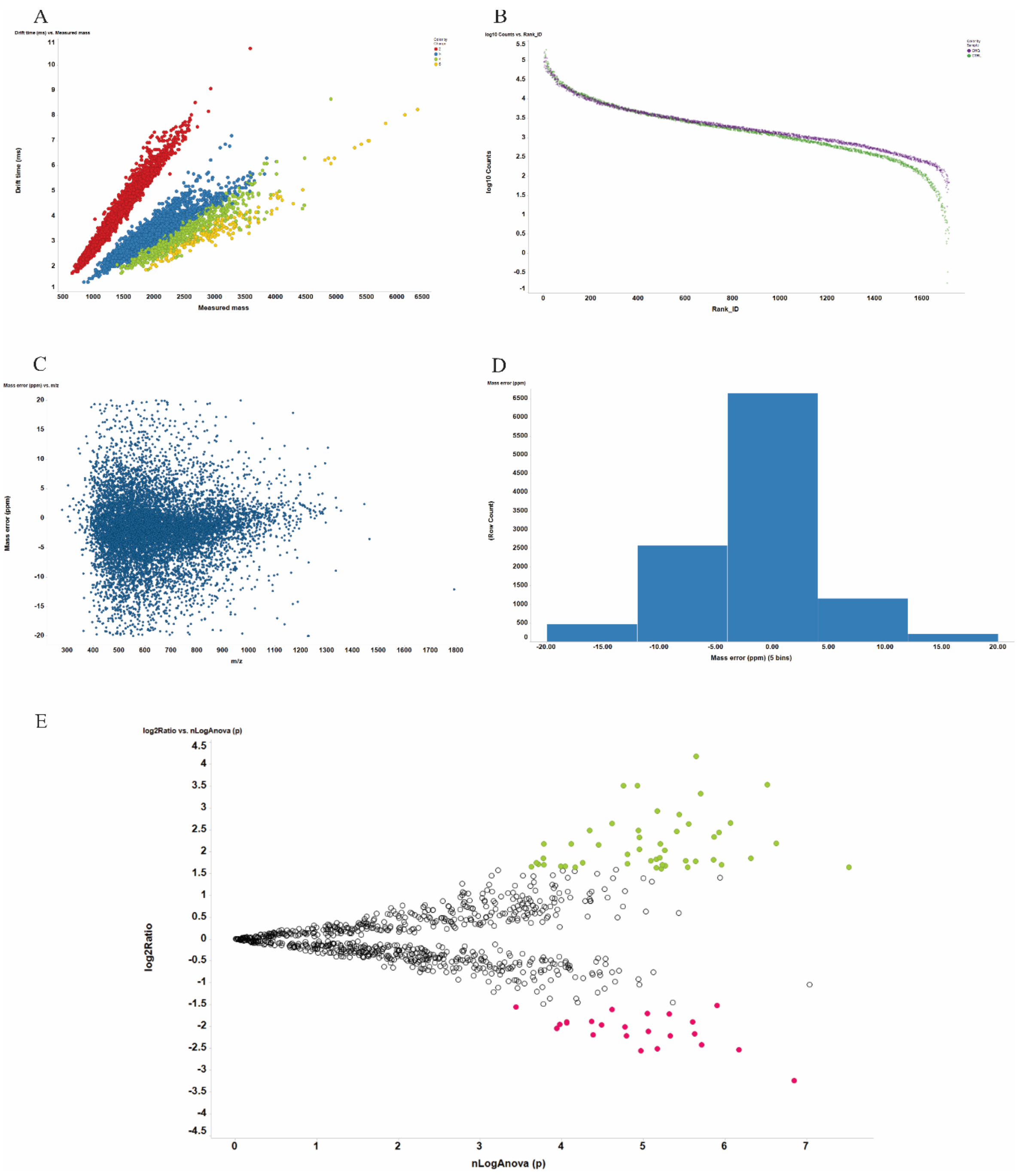

Figure S2: Mass spectrometry: analytical and physicochemical attributes for quality control (proteomics samples) – fom's. Fig.s A to E. Fig. A: peptide precursor mass (Da) and drift time (dt) separation of molecular species across different intrinsic charges and conformations ("chimericity") [64] discriminated by ion mobility separation – legend color by charge state; Red: [M + 2H]2+; Blue: [M + 3H]3+; Green: [M + 4H]4+; Yellow: [M + 5H]5+. B: Dynamic ranges of the quantified proteins in the study. Both conditions display a comparable profile due to similar protein quantitation fulfilled by the previous stoichiometry total ion count detection integrated area (σTIC) normalization. C: Mass error distribution of all peptide measurements, displaying the most dense and compact multitude of points across 0 ppm over the m/z range. D: Mass error distribution frequency displaying a normal distribution profile across 0 ppm. E: Volcano plot (log2fold vs. ANOVA (-log10p) of up (green), unchanged (black) and down-regulated (red) proteins.

Table S1: Characteristics of patients with septic shock

	P1	P2	P3	P4	P5
**Age**	71	68	51	61	32
**Gender**	M	M	F	M	M
**Days of ICU stay**	23	49	10	57	9
**Type of hospitalization**	Traumatic	Neurologic	Surgical	Clinical	Surgical
**Microbiological**	Gram-negative	Polymicrobial	Gram-negative	Polymicrobial	-
**Positive culture**	Hemoculture	Tracheal secretion	Hemoculture	Hemoculture	Negative
**Mechanical ventilation***	13	17	10	13	0
**AKF**	No	Yes	No	Yes	No
**Hemodialysis**	No	Yes	No	Yes	No
**SAPS3 score**	102	61	51	80	50
**%Mortality score**	92	38	19	74	17
**SOFA score**	16	11	14	11	17
**Clinical outcome**	Non-survive	Survivor	Non-survive	Survivor	Survivor

AKF, Acute Kidney Failure; SAPS3, Simplified Acute Physiology Score III; SOFA, Sequential Organ Failure Assessment; * Days of mechanical ventilation

Table S2: List of protein interactions

PROTEIN	INTERACTING PARTNERS
PPP1CB	RIPK3; SH2D4A; PPP1R11; TMEM33; BRCA1; PPP1R8; PPP1R2; IKBKG; PPP1R12C; PPP1R12B; RB1; SMARCB1; BCL2; PPP1R7; ZFYVE9; PTK2; RYR2; PPP1R12A; CDC34; WBP11; GRB2; MAX; CCND1; PPP1R15A; CDKN2A; PRKAR2A; PPP1R9B; AKAP11; CCND3; NCL; TLX1;
RIPK3	PPP1CB; MYH10; EPRS;
PDPK1	CDAN1; PKN1; MAPK8; AKT1; PRKCD; YWHAQ; PRKACA; PKN2; SGK1; STRAP; PRSS23; RPS6KB1; PTPRC; PRKCI; PTPRB; IRS1; TBL2; RPS6KA1; PTPN12; ITGB3; PTPRG; RPS6KA3; PRKCZ; PTK2B; PRKCE; PAK1; HSP90AA1; SLC9A3R2; SGK3; MTOR; PXN; AKT2; SND1; ILK; YWHAH; PNN; PRKCB; PTPN22; SRC; G3BP1; RPS6KB2; RALGDS; SGK2; PTPRK; PTPRJ; AKTIP; PTPRO; PA2G4; AKT3; CARD11;
EPRS	NELFCD; EEF1D; TFE3; IARS; ATG12; POU2F1; MAP3K14; RARS; MCC; ARL4D; IKBKE; MAPK13; AIMP2; RELB; HSP90AA1; DUS2; SYNCRIP; NFKBIB; IKBKB; MAP3K7; ETS1; RIPK3; NFKB2; NFKBIE; RELA;
FLNB	ATF7IP; NCK1; FBLIM1; GRB2; TSHR; NPHP1; PIK3R1; SMURF2; G3BP1; ITGB1; ITGB6; PLCG1; FLNA; OTUD1; PSEN2; CRK; PSEN1; GP1BA; ITGB3; PLEKHO1;
EFTUD2	DFFA; YWHAB; SNRNP40; USP39; PHLDA3; GSTK1; ARPC3; MYC; CD2BP2; ARRB2; AIRE; PTP4A3; PRPF8; SNRPB; RELA; YWHAG; RNPS1; MEPCE; TOP1; GOLM1; PRKAB1; NFKB2; SREK1; RPAP1; RPAP3; GPN1; RUVBL2;
PTPN1	LTK; JAK2; CRK; IGF1R; PIN1; CTNNB1; PIAS1; INSR; CDH2; GHR; CDK1; PDGFRB; STAT5A; GRB2; NTRK3; IRS1; BCAR1; MAPK1; CAV1; CSNK2A2; RRAS2; CLK1; BCR; TYK2; AKT1; STAT5B; ESR1; ACTN1; GSK3B; EGFR; TRPV6; CAPN2; NTRK2; NTRK1; GLRX; SRC; NFKBIA; CSNK2A1; CLK2; LAT;
ILK	ITGB3; PDPK1; S100A9;

ITGB3	ILK; FBLN2; PECAM1; NID1; AKT1; TLN1; KDR; VTN; CAPN1; DOK1; ITGAV; PDGFRA; PDPK1; DAB2; PTK2; NUMB; ITGB3BP; SHC1; FN1; THY1; ITGA2B; TNS2; TGM2; SRC; DAB1; FGA; FGG; CD36; THBS1; CIB1; FLNB; COL1A2; PTK2B; VWF; ANGPTL3; PXN; FLNA; PDGFRB; ITGA5; EPS8; P2RY2;
ANXA3	EMG1; UNC119; IGSF21; TP53; REG3A; UBR1;
COG4	COG7; COG5; COG2; COG1; COG3;
APOA1	ALB; KRT1; KRT9;
ALB	APOA1; SLX4; NPHS1; ST13; CDCP1; LUC7L2; ZNF232; AHSG; KIAA0232; DMWD; PDZRN4; SLA2; CUBN; CRB1; MYLK3; ATM; FCGRT; UIMC1; HP; APOC3; KIAA1551; QTRTD1; SCAF1; USP37; DICER1; RLF; PON1; C4A; SLC25A13; CABLES1; RANBP3; KLK3; APOE; SPATA31A7; KRT6A; RANBP2; KRT10; CROT; GRAP2; GFAP; LAT; AP4E1; CST3; GJC2; APOA2; SACS; RBP4; PALB2; AMPD3; ADRA1B; DCD; SGOL2; TF; CTAGE5; PLA2G4F; ITGA2; ORM1; SRGN; CRYAA; PRSS3; PPP2R2B; ETF1; TRAPPC11; APOC2; TTR; KRT5; AGA; KCNMA1; CACNA1I; TSC22D1; HPX; PLAG1; RYR2; BBC3; SH3BP5; KRT13; EXOC6; PDE4B; GABBR1; AMBP; LRP2; LDB3; ZNF292; PF4V1; CNOT1; THRAP3; SCN5A; PCDH1; DMD; DDB1; GSN; SLC9A8; F2; OBSL1; F7; CFB; CAMTA1; KRT16; KRT6B; ITIH1; FN1; ITGB5; DERL1; DGKG; CLCA2; CEP44; MYL4; SETX; IGDCC4; OR8D2; PPBP; OR2T6; TTPAL; NLRC4; DCC; CDC45; SORBS3; NR5A2; TLN2; TTN; CFH; CFD; PEG3; CTSL; FAM71E2; OR3A2; KRT9; GCN1; JARID2; SLC1A5; TIAM1; KRT1; SERPING1; NCOA3; PHC3; CHKB; AP1M1; FGA; HNF1A; PCED1B; SPAST; PRSS1; APOC1; YWHAG; KRT14;
SLX4	ALB; S100A8;
KRT1	APOA1; PRKCE; IVL; TANK; FANCA; LOR; MDM2; MBL2; CSTA; F12; FANCC; DSP; PI3; EVPL; PPP2R2B; CALB2; YWHAQ; KNG1; KRT5; APC; EGFR; ALB; CDH1; MAPK11;
HIST1H4A	CENPA; NASP; PAK1; HDAC4; GRB2; PTMA; HJURP; HIST1H3F; MAP3K7; MCM3; MAP3K3; SETD8; HIST1H1E; DNAJA2; HDAC5; H2AFX; ARRB1; RBBP7; HIST1H3E; ASF1A; MCM5; MCM4; HIST1H3B; MCM7; TBL1X; NPM1; MCM2; FKBP14; LIN54; HDAC6; HIRIP3; HIST1H3H; HIST1H3I; LIN37; HAT1; DAXX; DEK; RBBP4; HDAC1; KAT7; HIST1H3G; HIST1H3J; MAP3K1; MCM6; HIST1H3A; RB1; L3MBTL1; HIST1H3C; TNFRSF1A;
CCT8	PPP2CB; PPP2R2D; PPP4C; TCP1; PPP2CA; CTTNBP2; PPP2R2B; TBK1; IGBP1; STRN4; ACTB; STRN3; MYC; PPP2R4; MAP3K3; TRAF3IP3; TP63; RFWD2; PPP2R2C; STRN; MAP3K1; MAPK13; DOCK5; MOB4; CDK9; MEPCE; STK24; RELA; GPN1;
PSMC4	KRT33B; UCHL5; PSMD11; PSMD10; PSMC5; PAAF1; PSMD13; BAG2; RIOK3; TRIP6; USP14; PSMC2; PSMC3; PSMC6; HTR1E; PSMD1; PSMD2; ATG4C; PRKAB1; PAICS; EPB41; PSMD7; PSMD6; CMYA5; PSMC1; IKBKE; MYC; PSMD5; RORB; TRAF6;
NUDC	HLA-B; PAFAH1B1; VDAC1; HLA-C; PRKAB1; MAP3K3; LXN; BTRC; EIF6; ATG5; TIMP2; PLK1; TNIK; TNFRSF10D; PAK2; DGKE; FBXW11; WIPI2; EIF1B; ARF6; MAPK13; ELF3; VHL;
S100A9	S100A8; PPP2R2B; USF2; TAGLN; ARRB2; EGFR; PAK7; ASB3; ARRB1; NUAK1; CFTR; PPP2R1A; ILK;
S100A8	S100A9; NCF2; MOB1A; LRIF1; PRMT1; C14orf1; PPIA; PDCD11; CHGB; SLX1B; IGSF21; MAP3K3; ASB3; RAB17; NUAK1; SLX1A; USF2; PPM1B; CDK2; CEACAM3; UNC119; KLC2; SLX4; PPP2R2B; TP53; LRRK1; TUBA4A; TRAF3IP1; GDF9; NFKBIB; RIF1; DMWD;
EIF1B	ACP1; NUDC; HMGB2;
ACP1	EIF1B; IKBKE; EPHB1; NR3C1; FYN; TNIK; CTNNB1; SFMBT1; EPHB2; SPTAN1; FNBP1L; EPB41; INSR; PAK2; EIF6; EPHA2; FABP4; LCK; VHL; ZAP70; KDR; MRPL20;
MYO5A	BMF; RAB27A; MYC; MLPH; TRIM2; DYNLL2; DYNLL1; SMAD2; SHANK2; PRPH; RAB11A; TRIM3; DLGAP1; NEFL;
FOS	ZNF133; MATR3; TLN2;
ZNF133	FOS; MDM2;
ANAPC5	SMURF1; CDC16; ANAPC16; CDC20; CDC23; ANAPC13; CREBBP; ANAPC1; ZBTB16; CDC27; ANAPC2; PABPC1; ANAPC7; ANAPC4; EP300; APC2; MED19; FZR1; CDT1; SMURF2; BUB3; CDC26; TGFBR1; BUB1B;
CRK	PTPN1; FLNB;

MYH10	ARRB2; COPS5; TNFRSF10D; PRKAB1; SVIL; PBX1; TNFRSF1A; IKBKE; RELA; PAK2; GRB2; MYH9; IKBKG; LLGL1; BCAP31; TNFRSF1B; EPB41; MCC; RIPK3; MAP3K1; S100A4; IKBKB; MARK4; USP45; RIPK2; CHUK; MAP3K3;
ARRB2	MYH10; S100A9; EFTUD2;
IKBKE	ACP1; MYH10; EPRS; PSMC4;
AKT1	PDPK1; ITGB3; KRT10; PTPN1; DLC1;
MATR3	RELA; NFKB2; GFI1B; GSTK1; NR4A1; CDK9; GRB2; MYC; PCBP1; DISC1; EGFR; FOS; TTF2; RUVBL2; MAP3K3; HNRNPK; MEPCE; PPP2R2B; H2AFX;
RELA	MATR3; EFTUD2; MYH10; CCT8; EPRS;
PRKCE	KRT1; PDPK1;
LZTR1	TUBGCP4; BMPR1B;
YWHAQ	PDPK1; KRT9; KRT1;
TANK	KRT9; KRT1;
KRT9	TANK; PPP2R2B; MDM2; TRAF3IP1; APC; SH3GL3; CDH1; YWHAQ; ALB; APOA1;
MLH3	MLH1; MSH4;
LTF	LRP1; MUC7; CD14; SGK1; CP; CDK5RAP3; CALM1; ITLN1; CEL; LCN1; LYZ;
LRP1	LTF; CTSG;
CTNNB1	PTPN1; ACP1;
NR3C1	ACP1; HMGB2;
TLN2	PIP5K1C; FOS; ALB;
HMGB2	POU2F2; PRKDC; PGR; RAG1; CSNK1A1; GZMA; POU3F1; PON2; SET; NFKB1; EIF1B; AR; POU2F1; APEX1; TP53; TRAF6; NR3C1; POU5F1;
C8B	C5; C8A; CLU;
ATP6V1B1	STX1A; ACTN4; SLC9A3R1;
CHTF8	DSCC1; EIF4EBP1; CHTF18; PCNA; RFC2; RFC3;
SGK1	PDPK1; LTF;
UNC119	ANXA3; S100A8;
DMWD	ALB; S100A8;
PPP2R2B	S100A9; KRT9; KRT10; CCT8; ALB; S100A8; KRT1; MATR3;
POU2F1	EPRS; HMGB2;
TNC	NCAN; FN1; EGFR; CNTN1; PTPRB; ITGA5;
KDR	ITGB3; ACP1;
CTSG	C3; SERPINA1; PPBP; SELPLG; SDC1; CASP7; CXCL12; GP1BA; F2RL2; CAMP; KNG1; F2R; IGFBP3; PARP1; F5; CXCR4; VCAM1; SERPIND1; THBS1; SERPINB13; LRP1; F2RL1; SERPINA3; AGT;
IKBKG	PPP1CB; MYH10;
PAK1	HIST1H4A; PDPK1;
SMAD2	ZEB2; MYO5A;
ZEB2	SMAD2; SMAD9; SMAD5; SMAD1; SMAD3; COPS6; CTBP2; SCHIP1; CTBP1;
TNFRSF10D	MYH10; NUDC;
GRB2	FLNB; HIST1H4A; PTPN1; MATR3; MYH10; PPP1CB;
PRKAB1	MYH10; NUDC; PSMC4; EFTUD2;

TRAF3IP1	KRT10; KRT9; S100A8;
KRT10	TRAF3IP1; PPP2R2B; TJP1; EVPL; AKT1; RPS9; ALB; PRKCZ; SMAD3; MDM2; NFKB2; GLE1; MME;
INSR	PTPN1; ACP1;
GSTK1	EFTUD2; MATR3;
TNIK	ACP1; NUDC;
MYC	MYO5A; EFTUD2; MATR3; CCT8; PSMC4;
USF2	S100A9; S100A8;
HLA-C	NUDC; KIR2DL2;
NFKB2	MATR3; HIST1H2BB; KRT10; EFTUD2; EPRS;
PTPRB	PDPK1; TNC; NRCAM;
RB1	PPP1CB; HIST1H4A;
IRS1	PDPK1; PTPN1;
MAP3K3	NUDC; HIST1H4A; S100A8; CCT8; MATR3; MYH10;
PDGFRB	PTPN1; ITGB3;
MAP3K7	HIST1H4A; EPRS;
EVPL	KRT10; KRT1;
EGFR	S100A9; TNC; PTPN1; MATR3; KRT1;
SMURF2	FLNB; ANAPC5;
NRCAM	CNTN2; MAGI3; MACF1; HSPA12A; ANK2; NFASC; PTPRB;
G3BP1	FLNB; PDPK1;
LCN2	MMP2; MMP9; HGF; LRP2;
TUBGCP5	TUBG1; TUBGCP3; MZT1; MZT2B;
CDK9	MATR3; CCT8;
MDM2	KRT1; ZNF133; KRT9; KRT10;
EIF6	NUDC; ACP1;
FN1	TNC; ITGB3; ALB;
SMAD3	ZEB2; KRT10;
KIR2DL2	HLA-C; CD93;
IGSF21	S100A8; ANXA3;
AIRE	EFTUD2; HIST1H2AC;
HIST1H2BB	TNFRSF1A; UHRF1; NFKB2;
TNFRSF1A	HIST1H2BB; MYH10; HIST1H4A;
MCC	EPRS; MYH10;
HIST1H2AC	TFAP2B; AIRE;
PPBP	CTSG; ALB;
ASB3	S100A8; S100A9;
PRKCZ	PDPK1; KRT10;
PTK2B	PDPK1; ITGB3;
EPB41	ACP1; PSMC4; MYH10;
DEFA1	PFDN1; DEFA3; SERPINF2; C1QB;

YWHAG	EFTUD2; ALB;
LAT	ALB; PTPN1;
PTK2	PPP1CB; ITGB3;
H2AFX	HIST1H4A; MATR3;
PAK2	MYH10; ACP1; NUDC;
ARRB1	HIST1H4A; S100A9;
FLNA	FLNB; ITGB3;
NUAK1	S100A8; S100A9;
RYR2	PPP1CB; ALB;
MAPK13	EPRS; CCT8; NUDC;
MEPCE	EFTUD2; CCT8; MATR3;
SRC	ITGB3; PTPN1; PDPK1;
CEACAM6	CEACAM8; CEACAM1;
HSP90AA1	PDPK1; EPRS;
KRT5	ALB; KRT1;
FGA	ITGB3; ALB;
CP	LTF; MPO;
VHL	ACP1; NUDC;
GP1BA	CTSG; FLNB;
DLC1	TNS3; AKT1;
TP53	S100A8; ANXA3; HMGB2;
PXN	PDPK1; ITGB3;
LRP2	ALB; LCN2;
THBS1	ITGB3; CTSG;
KNG1	CTSG; KRT1;
MAP3K1	MYH10; CCT8; HIST1H4A;
SACM1L	COPG1; COPB2; COPA;
RUVBL2	MATR3; EFTUD2;
NFKBIB	EPRS; S100A8;
APC	KRT9; KRT1;
IKBKB	EPRS; MYH10;
CDH1	KRT9; KRT1;
ITGA5	TNC; ITGB3;
TRAF6	HMGB2; PSMC4;
GPN1	EFTUD2; CCT8;

Table S3: The major proteins correlation cell function

Protein	Phenotype	Activation	Phagocytosis	Migration	Senescence	Death	Viability

ANXA3			■	■		■	
FLNB				■		■	
ZEB2	■			■		■	
EPRS	■	■				■	
RETN		■					
LCN2	■			■		■	
PDPK1					■		
NNMT	■					■	■
RNASE3					■		■
DLC1	■			■	■		
SACMIL	■						■
TLN2				■			
LTF	■	■	■	■		■	
PPP1CB	■						
S100A8	■	■	■	■		■	
PTPN1						■	
KLB	■				■	■	
CTSG	■	■	■	■			
MPO	■	■	■	■		■	
EFTUD2							■
NRCAM	■			■		■	
S100A9	■	■	■	■		■	
MATRA3						■	
CEACAM 6	■		■			■	
KIR2DL2			■				
NUDC	■			■		■	
SH3TC2				■			
HIST1H2AC	■						
ACP1	■	■		■		■	
ITGB3	■	■	■				
TNC	■	■	■	■		■	
ALB	■	■	■	■			
HMGB2	■	■					■
MYH10	■			■		■	
MYO5A	■		■	■		■	
ESRP2	■			■			
PSMC4						■	
KRT1	■	■					
KRT10	■	■				■	
ZFHX2	■					■	
LZTR1						■	
CLCN4	■			■			
CCT8				■			
ATP13A3					■		
ATAD2B	■						
KRT9	■						
